# Efficacious resolution of a rectal subepithelial cold abscess via endoscopic submucosal excavation

**DOI:** 10.1055/a-2194-4387

**Published:** 2023-11-14

**Authors:** Xinyue Mao, Silin Huang, Bo Li, Suhuan Liao, Ronggang Zhang, Jun Cai, Heng Yu

**Affiliations:** Department of Gastroenterology, South China Hospital, Medical School, Shenzhen University, GuangDong, China

A 39-year-old woman underwent a colonoscopy subsequent to episodes of hematochezia, and this revealed the existence of a rectal subepithelial mass, approximately 18 × 8 mm in size, covered with smooth, normal mucosa. Endoscopic ultrasound suggested that the lesion originated from the submucosal layer, locally infringing upon the muscularis propria, and that it showed homogeneous hypoechoic changes, clear boundaries, and was growing toward the lumen ( Fig. 1 a ). Computed tomography revealed a roundish elevated lesion with an envelope in the rectum, which exhibited significant enhancement of the envelope following contrast administration, but showed no evidence indicating lymphatic or organ metastasis ( Fig. 1 b ). The patient was hospitalized and underwent endoscopic excision of the lesion ( Video 1 ). During the procedure, the lesion was found to be a tough mass, mostly located in the submucosal layer, and when submucosal dissection was performed, the underside of the mass was seen to intrude into the muscularis propria in a strip-like manner. Consequently, the myofibers adjacent to the mass were excised, revealing the root of the mass, which was completely dissected. The procedure was completed without any perforation of the wound, and closure was achieved employing nylon suture and metallic clips ( Fig. 2 ). Postoperative antibiotics were administered to prevent infection, and the patient remained free of symptoms such as fever, abdominal pain, or hematochezia. Histopathologic study confirmed a deep encapsulated pyogenic inflammation, characterized by an accumulation of neutrophils, lymphocytes, plasma cells, and histiocytes forming an abscess ( Fig. 3 ).

**Fig. 1 a:**
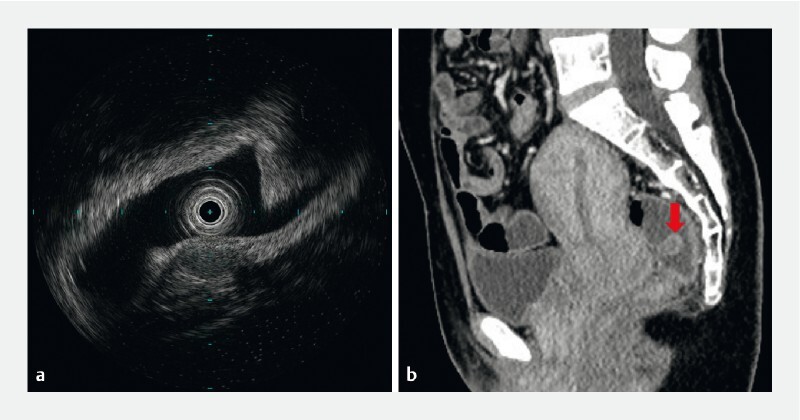
Endoscopic ultrasound suggested that the lesion originated from the submucosal layer, and that it locally involved the muscularis propria. **b** Enhanced computed tomography suggested enhancement of the subepithelial mass envelope.

**Video 1** Endoscopic submucosal excavation in the management of a rectal subepithelial cold abscess.


**Fig. 2:**
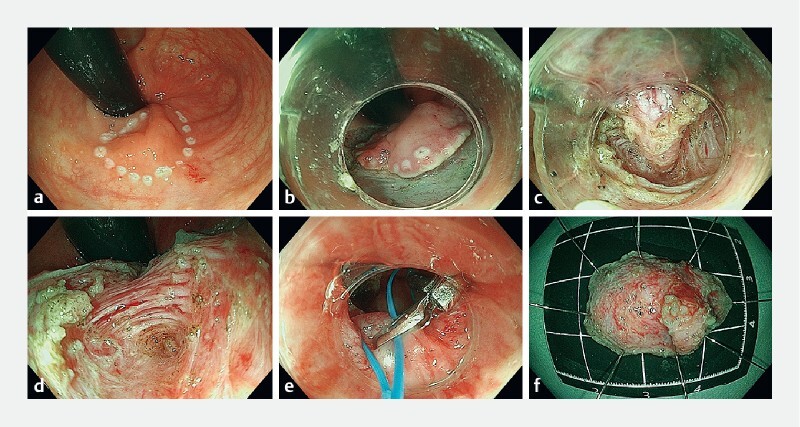
Endoscopic submucosal excavation: operating procedure. **a** Circumferential marking. **b** Mucosal incision. **c** Dissection along the envelope. **d** Wound after lesion excision. **e** Closure of the wound. **f** Excised mass.

**Fig. 3:**
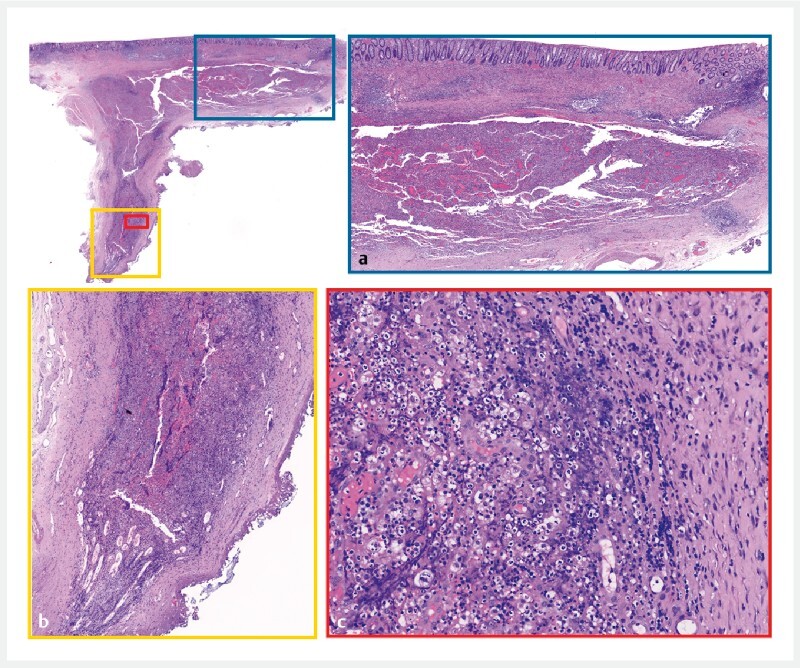
Histopathology. **a, b** The lesion was a deep encapsulated pyogenic inflammation. **c** Accumulation of neutrophils, lymphocytes, plasma cells, and histiocytes forming an abscess.

Redness, swelling, warmth, pain, and impaired function are the typical manifestations of acute inflammation 1 2 . An abscess without these acute inflammatory hallmarks is designated a cold abscess 3 4 5 . Cold abscesses within the digestive tract are exceedingly rare. To the best of our knowledge, this represents the first reported instance of a rectal cold abscess mimicking a subepithelial mass, which was effectively managed through endoscopic submucosal excavation.

Endoscopy_UCTN_Code_CCL_1AD_2AI

## Notes

Endoscopy E-Videos

https://eref.thieme.de/e-videos
*E-Videos* is an open access online section of the journal *Endoscopy* , reporting on interesting cases and new techniques in gastroenterological endoscopy. All papers include a high-quality video and are published with a Creative Commons CC-BY license. Endoscopy E-Videos qualify for HINARI discounts and waivers and eligibility is automatically checked during the submission process. We grant 100% waivers to articles whose corresponding authors are based in Group A countries and 50% waivers to those who are based in Group B countries as classified by Research4Life (see: https://www.research4life.org/access/eligibility/ ). This section has its own submission website at https://mc.manuscriptcentral.com/e-videos

## References

[1] Gaertner W B Burgess P L Davids J S The American Society of Colon and Rectal Surgeons clinical practice guidelines for the management of anorectal abscess, fistula-in-ano, and rectovaginal fistula Dis Colon Rectum 2022 65 964 985 35732009 10.1097/DCR.0000000000002473

[2] Jan I A Durell J Lakhoo K Common bacterial infections of surgical importance Cham Springer International Publishing 2020 155 164

[3] Muramatsu K I Nagasawa H Murai Y Non-tuberculosis cold abscess Am J Emerg Med 2020 38 INF INF 10.1016/j.ajem.2020.04.096 32444294

[4] Adepoju O J Nweke M C Soneye O Y Unilateral hypoplastic pelvic ectopic kidney presenting as a cold abscess: a case report Niger J Surg 2021 27 55 58 34012243 10.4103/njs.NJS_27_19 PMC8112373

[5] McFarland J R 3rd Branch D Gonzalez A L5 fracture dislocation secondary to cold abscess treated by posterior corpectomy with expandable cage placement Cureus 2020 12 e8756 32714694 10.7759/cureus.8756 PMC7377670

